# Immunomodulation by *Trypanosoma cruzi*: Toward Understanding the Association of Dendritic Cells with Infecting TcI and TcII Populations

**DOI:** 10.1155/2014/962047

**Published:** 2014-10-13

**Authors:** Thiago Alvares da Costa, Marcos Vinicius Silva, Maria Tays Mendes, Tamires Marielem Carvalho-Costa, Lara Rocha Batista, Eliane Lages-Silva, Virmondes Rodrigues, Carlo Jose Oliveira, Luis Eduardo Ramirez

**Affiliations:** ^1^Postgraduate Course of Tropical Medicine and Infectology, Laboratory of Immunology, Federal University of Triângulo Mineiro, Avenida Getúlio Guaritá S/N, 38015-050 Uberaba, MG, Brazil; ^2^Postgraduate Course of Tropical Medicine and Infectology, Laboratory of Parasitology, Federal University of Triângulo Mineiro, Uberaba, MG, Brazil

## Abstract

Dendritic cells (DCs) are major immune components, and depending on how these cells are modulated, the protective host immune response changes drastically. *Trypanosoma cruzi* is a parasite with high genetic variability and modulates DCs by interfering with their capacity for antigen recognition, migration, and maturation. Despite recent efforts, the association between DCs and *T. cruzi* I (TcI) and TcII populations is unknown. Herein, it was demonstrated that AQ1.7 and MUTUM TcI strains present low rates of invasion of bone marrow-derived DCs, whereas the 1849 and 2369 TcII strains present higher rates. Whereas the four strains similarly induced the expression of PD-L1, the production and expression of IL-10 and TLR-2, respectively, in DCs were differentially increased. The production of TNF-*α*, IL-12, IL-6, and CCL2 and the expression of CD40, CD80, MHC-II, CCR5, and CCR7 changed depending on the strain. The 2369 strain yielded the most remarkable results because greater invasion correlated with an increase in the levels of anti-inflammatory molecules IL-10 and PD-L1 but not with a change in the levels of TNF-*α*, MHC-II, or CD40 molecules. These results suggest that *T. cruzi* strains belonging to different populations have evolved specific evasion strategies that subvert DCs and consequently the host response.

## 1. Introduction

*Trypanosoma cruzi*, the causative agent of Chagas disease, presents high genetic and biologic variability, and the different strains described can differ in their morphology, tissue tropism, virulence and pathogenicity, susceptibility to chemotherapeutic agents, and antigenic composition, among other features [1]. After an extensive literature review and assessment of biological, biochemical, and molecular phylogenetic markers from different strains, researchers currently classify* T. cruzi* into six discrete genetic subdivisions, or “discrete typing units” (DTUs), designated as* T. cruzi* I (TcI), TcII, TcIII, TcIV, TcV, and TcVI [2, 3]. TcI and TcII DTUs are the first ancestral groups, and the other groups were generated from these two DTUs. The strains from TcI and TcII are considered the major causative agents of Chagas disease all over the world and especially in South America, where the disease and the two groups are more prevalent [2, 3]. The TcI strains are mainly relevant to acute infections and severe cases of acute myocarditis [4, 5], and the strains from TcII and TcIV are more relevant to the cardiac and digestive forms of Chagas disease. The DTU TcV is most commonly found in the congenital transmission of Chagas disease, and TcIII is considered rare in human infections [2].

It is widely accepted and demonstrated that, beyond the strain that causes the disease, the differences in the clinical and epidemiological features of Chagas disease are also related to the host immune response [6]. Thus, the establishment of* T. cruzi* infection depends on a series of events involving interactions between the parasite and the host. First, the parasite infects the host cell, either by active penetration [7] or by* T. cruzi*-host cell phagocytosis [8]. Subsequently, the parasite develops and spreads to these cells and may also modulate the cells' biology. If the infected cells are immune cells, one of the main features of* T. cruzi* is its ability to modulate these immune defense effectors' mechanisms.

One of the main cell types of the host immune response that is targeted by* T. cruzi* is the dendritic cells (DCs) [9]. DCs are antigen-presenting cells that are derived from bone marrow precursors and that participate in the activation of the innate and adaptive immune responses. Once differentiated, DCs migrate to peripheral tissues, recognize, capture, and process antigens at these sites (e.g., via toll-like receptors (TLRs)), and become activated. Once activated, the cells migrate via CCR7 to secondary lymphoid organs, where they present the antigens to T cells and produce cytokines such as IL-12, IL-10, TNF-*α*, and IL-6, which contribute to the activation and differentiation of antigen-specific T lymphocytes. In the DC-T cell interaction in lymphoid organs, the major histocompatibility complex (MHC I or MHC II) interacts with the T cell receptor (TCR), and costimulatory molecules such as CD40, CD80, CD83, and CD86 interact with their respective costimulatory ligands on the T cells, providing necessary molecular signals that result in the proliferation and differentiation of naive T cells [10, 11].

Protozoa such as* T. cruzi* modulate the function of DCs by interfering with these cells' recognition, migration, maturation, and antigen presentation. It is known that* T. cruzi* inhibits the expression of MHC II, CD40, CD80, and CD86, hampers the production and secretion of IL-12, TNF-*α*, and IL-6 and increases the production of the cytokine IL-10, and inhibits the presentation of antigens in murine and human DCs in vitro [9, 12–15]. Because the cited studies showed that the DCs had an anti-inflammatory profile, certain authors classify these cells as regulatory DCs [16]. Corroborating the in vitro findings, in vivo experiments have also demonstrated that* T. cruzi* is able to impair many aspects of DC biology. During acute infection, splenic DCs' migration and expression of the costimulatory molecule CD86 are inhibited in mice infected with* T. cruzi* [17]. In addition to the inhibition of costimulatory molecules, it is known that* T. cruzi* can induce the expression of coinhibitory molecules, such as PD-L1 and its ligand, PD-1. These molecules can trigger inhibitory signals to T cells, which culminate in less activation and more apoptosis of T lymphocytes [18].

Chagas disease presents diverse clinical manifestations, and such diversity is suggested to be dependent on the heterogeneity among* T. cruzi* strains, their evasion mechanisms, and even variation in the host immune response itself [19, 20]. Despite all of this information and the vast literature on the anti-*T. cruzi* immune response, the majority of studies published so far have only evaluated a single strain of this parasite. Given the diversity of the strains, the differential ability of each to generate disease, and the importance of DCs to mounting a successful immune response against* T. cruzi*, we sought to evaluate whether different strains of TcI and TcII differentially modulate the biology of DCs.

## 2. Materials and Methods

### 2.1. *T. cruzi* Strains

The blood trypomastigote forms of the* T. cruzi* strains used in this study were obtained from the collection of strains of the Discipline of Parasitology, Federal University of Triângulo Mineiro, and they were cryopreserved in liquid nitrogen at −186°C. The four selected strains were as follows: AQ1.7, isolated from* Triatoma sordida* captured in the city of Agua Quente, BA; MUTUM, isolated from* Panstrongylus megistus* captured in the city of Uberaba, MG; 1849, isolated from an HIV+ patient presenting the cardiodigestive form of Chagas disease; and 2369, isolated from an HIV+ patient presenting the neurological form of Chagas disease. The AQ1.7 and MUTUM strains are classified as belonging to the DTU TcI [21], and strains 1849 and 2369 are classified as belonging to the DTU TcII (E. Lages-Silva, personal communication). The epimastigotes were thawed and placed in a culture of LLC-MK2 cell monolayers in LIT medium. The cells were incubated at 37°C in a humidified atmosphere containing 5% CO_2_, and the medium (DMEM, Sigma, St. Louis, MO, USA) was periodically replaced until blood trypomastigotes were observed in the culture supernatant.

### 2.2. Experimental Animals, Reagents, and Chemicals

Female C57BL/6 mice (6–8 weeks of age), used for obtaining DCs, were bred and maintained under standard conditions in the animal facilities of the Institute of Biological and Natural Sciences, Federal University of Triângulo Mineiro, Uberaba, MG, Brazil. All animal experiments were performed in accordance with a protocol (protocol number 299) approved by the University Federal of Triângulo Mineiro Institutional Animal Care and Use Committee. Ultrapure* Escherichia coli* 0111:B4 lipopolysaccharide (LPS) was purchased from InvivoGen (San Diego, CA, USA). Granulocyte macrophage-colony stimulating factor (GM-CSF), a selective inductor of DCs, was purchased from PeproTech (Rocky Hill, NJ, USA). The doses of LPS and GM-CSF used in this work were determined based on the manufacturers' recommendations and/or our own dose-response studies (data not shown). Antibodies for flow cytometry and cytokine kits (OptEIA ELISA sets) were purchased from eBioscience (San Diego, CA) or BD Biosciences (San Jose, CA). All experiments were replicated twice, with triplicates for each parameter evaluated.

### 2.3. Dendritic Cells

DCs were generated from C57BL/6 WT mice as described previously [22], with certain modifications. Briefly, bone marrow cells from femurs and tibias were cultured in 10 mL of complete RPMI medium (RPMI 1640 medium with 10% heat-inactivated FBS, 2 mM L-glutamine, 100 IU penicillin, 100 *μ*g/mL streptomycin, and 0.05 mM 2-mercaptoethanol) and 25 ng/mL GM-CSF. At day 0, the cells were seeded at 2 × 10^6^ per 100 mm Petri dish. At days 3 and 6, another 10 mL of complete medium containing 50 ng/mL GM-CSF was added to the 10 mL already present in the dishes. The differentiated cells harvested on days 6-7 of culture were analyzed for the expression of CD11c and CD11b by flow cytometry. Only cultures of DCs that had percentages of differentiation above 75% (CD11c+CD11b+) were used in this work.

### 2.4. *T. cruzi* Invasion Assay

To assess the infectivity of the four strains, bone marrow-derived DCs were resuspended in 24-well plates at a concentration of 2 × 10^5^ cells/well. The trypomastigote forms of* T. cruzi,* obtained from the culture of LLC-MK2 cells, were incubated with phosphate-buffered saline (PBS) plus 1 nM CFSE for 5 min in the dark for staining. Afterward, the parasites were added to the DC cultures for 18 h at a parasite-cell ratio of 3 : 1. The cells were then collected, washed to remove parasites that had not invaded the cells, and analyzed using a FACSCalibur cytometer (Becton Dickinson, Mountain View, CA, USA). CD11c+CFSE+ cells (50,000 events/tube) were analyzed using CellQuest 5.1 and FlowJo 10 (TREESTAR, Ashland, OR, USA) software.

### 2.5. Apoptosis Assay

Bone marrow-derived DCs were distributed in 24-well plates at a concentration of 2 × 10^5^ cells/well, and* T. cruzi* trypomastigotes from the different strains were added to the cultures for 18 h at a parasite-cell ratio of 3 : 1. Afterward, the DCs were washed with PBS, and annexin V-fluorescein isothiocyanate (annexin V-FITC; 2.5 *μ*g/mL) and propidium iodide (PI; 2.5 *μ*g/mL) staining was performed according to the manufacturer's specifications (BD Biosciences). A minimum of 30 × 10^4^ DCs per* T. cruzi* strain infection were analyzed by fluorescence-activated cell sorting (FACS), and Annexin V^−^  PI^−^ cells were considered as viable cells. The data acquired using a FACSCalibur cytometer were analyzed using CellQuest 5.1 and FlowJo 10 software.

### 2.6. Cytokine Assay

Bone marrow-derived DCs w0065re incubated with the AQ1.7, MUTUM, 1849, or 2369 strain of* T. cruzi* or with LPS for 18 h. LPS was only used as a positive control in the experiment. Next, the supernatant of each culture was collected and used for the measurement of cytokine and chemokine levels. Measurements of the levels of the cytokines IL-12p40, TNF-*α*, IL-10, and IL-6 and of the chemokine CCL2 were performed using a specific solid-phase sandwich enzyme-linked immunosorbent assay (ELISA). For the measurement of IL-6 and IL-12p40 levels, samples were diluted 10 and 20 times, respectively. BD OptEIA ELISA sets were used according to the manufacturer's instructions (BD Biosciences). The concentrations of the cytokines were calculated by linear regression on the absorbance values obtained for the recombinant cytokines and expressed as pg/mL. The sensitivity of the tests ranged from 2 to 20 pg/mL. None of the culture supernatants was thawed more than once.

### 2.7. Expression of DC Surface Markers by DCs

To evaluate the effect of the different strains of* T. cruzi* on the activation of DCs, we exposed DCs to the four strains of* T. cruzi* or LPS (positive control). After incubation, the DCs were collected for flow cytometric analysis. Briefly, after incubation with Fc block for 30 min on ice, the collected DCs were washed with 1X PBS and cultured with the following antibodies for 30 min: PE-CY7-conjugated anti-CD11c; PE-conjugated anti-MHC II, anti-CCR5, anti-PD-L1, or anti-TLR2; or FITC-conjugated anti-CD11b, anti-CD40, anti-CD80, anti-CD83, anti-CD86, anti-CCR7, or anti-TLR4. After washing the cells twice in PBS, data acquisition was performed using a FACSCalibur with CellQuest 5.1 software and analyzed with FlowJo software. The acquired results are expressed as the mean fluorescence intensity (MFI, arbitrary unit standardized for all experiments based on negative controls) of positive cells and/or the relative frequency (%) obtained with each antibody within the studied gates [23]. Appropriate isotype-matched irrelevant mAbs were used as negative controls for each DC molecule analyzed.

### 2.8. Statistics

A statistical analysis was performed using the program GraphPad Prism 5.0 (GraphPad Software, San Diego, CA, USA). Continuous variables are expressed as the mean ± standard deviation. The Mann-Whitney test was used for the comparison of two independent groups. Differences were considered significant when *P* < 0.05 (5%).

## 3. Results

### 3.1. The Infectivity of* T. cruzi* in DCs Is Strain Dependent

**Figure 1:**
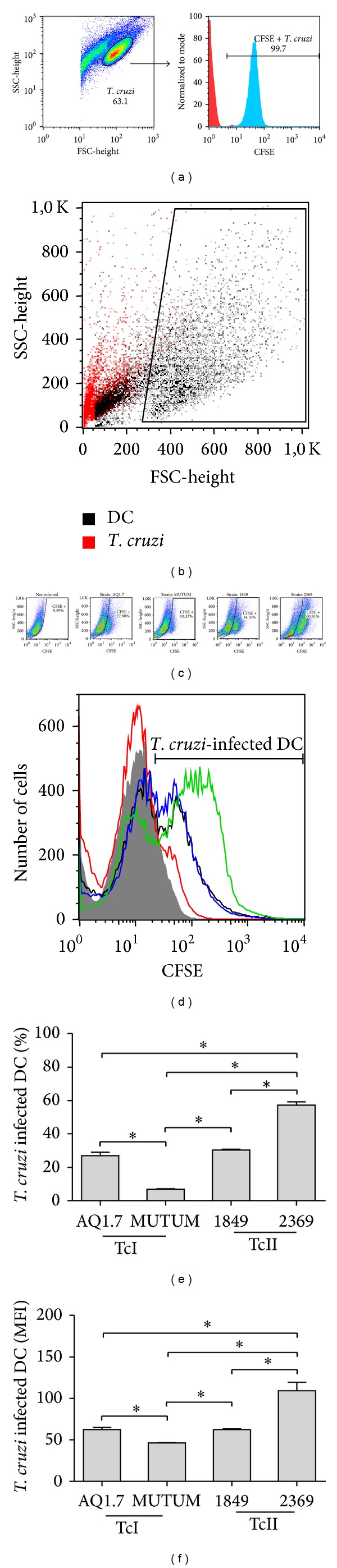
DC infection rates depend on the infective* T. cruzi* strain. Murine bone marrow-derived DCs were incubated for 18 h with different CFSE-labeled* T. cruzi* strains (1 nM CFSE; MOI 3 : 1), and fluorescent DCs were quantified by flow cytometry. (a) Schematic representation of the gating strategy and determination of* T. cruzi* CFSE staining (>99.5% for all strains). (b) DCs and* T. cruzi* present different FSC × SSC patterns, allowing the determination of DC infection without nonintracellular* T. cruzi* interference. (c) Representative dot plots for* T. cruzi*-infected DCs (CFSE+; left to right: noninfected, AQ1.7, MUTUM, 1849, and 2369). (d) Representative histogram of CFSE+ DCs infected with different* T. cruzi* strains (d), the % of infected DCs (e), and the parasite load per DC based on the MFI of positive cells (f).

In general terms, the experiments demonstrated that the strains AQ1.7, MUTUM, 1849, and 2369 have different capacities to infect DCs (Figure 1). In Figure 1(a), we present a scheme demonstrating* T. cruzi* CFSE staining that had a positivity of nearly 99%, which is representative of all strains studied. Furthermore, we demonstrated that DCs and* T. cruzi* present different FSC × SSC patterns, allowing the determination of DC infection without nonintracellular* T. cruzi* interference (Figure 1(b)). Representative dot plots (Figure 1(c)) and the percentages and the MFIs of CFSE+ DCs (Figure 1(d)) demonstrated that the MUTUM strain showed a lower rate of invasion (10.33%), whereas strain 2369 had the highest rate of infectivity (60.81%). The strains AQ1.7 and 1849 had intermediate rates of infection compared with the MUTUM and 2369 strains; in particular, these strains showed infectivity rates of 22.88 and 34.18, respectively (Figures 1(c) and 1(d)). As can be observed in Figure 1(d), both the percentage and the MFI of the infectivity of the different strains of* T. cruzi* presented great similarity. It is also possible to observe that, even within the same DTU (TcI: AQ1.7 compared with MUTUM; TcII: 1849 compared with 2369), the percent infectivity may vary significantly (Figure 1(d)).

### 3.2. The Invasion of DCs by Different* T. cruzi* Strains Does Not Alter DC Viability

**Figure 2:**
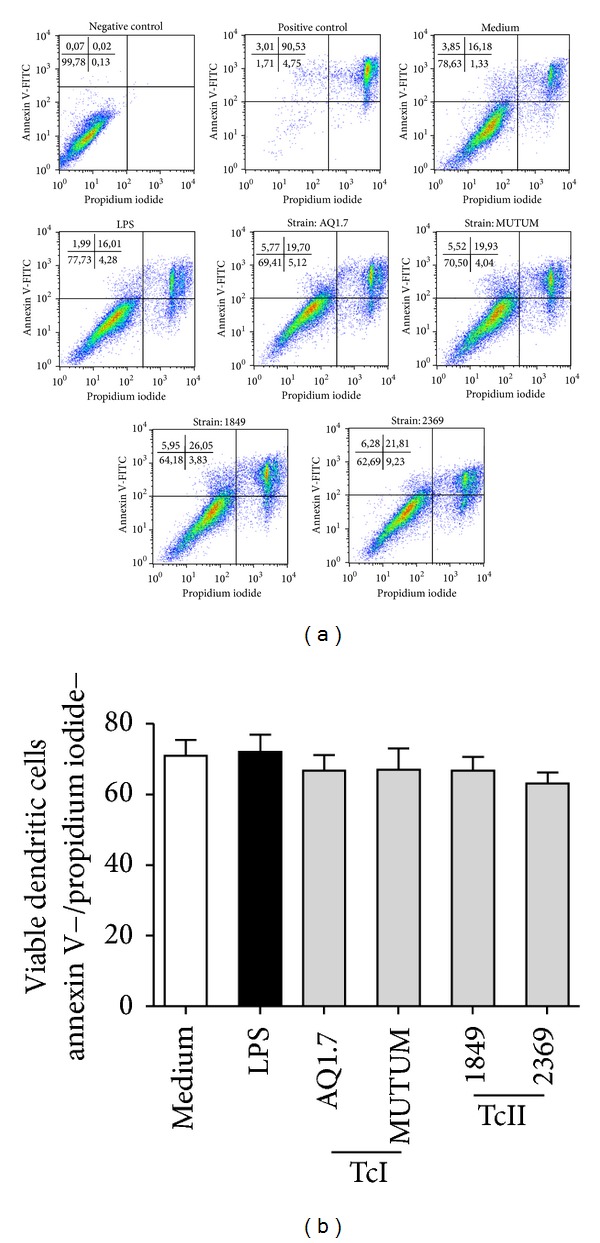
*T. cruzi* strains do not alter DC viability. Murine bone marrow-derived DCs were incubated for 18 h with no stimulation (medium), LPS (100 ng/mL), or different* T. cruzi* strains (MOI 3 : 1), and viable cells were identified by a lack of annexin V or PI staining. (a) Dot plots of representative samples showing the % of viable cells (Annexin V-FITC- and/or PI-DCs, left quadrant). Negative control: unstained DCs. Positive control: previous to annexin V and PI staining, the DCs were maintained for 30 min at 57°C. (b) The % of viable DCs. The bars represent the mean, and the vertical lines represent the standard error; Student's *t*-test or Mann-Whitney test.

Knowing that all* T. cruzi* strains used in this study were able to infect DCs, although at very different rates, we performed an assay to determine whether differentially infected DCs would have altered viability after parasite invasion. The apoptosis assay demonstrated that the different strains used were not capable of altering the viability of the DCs after infection (Figure 2). As we can see in Figures 2(a) and 2(b), both the representative dot plots and the percentages of viable DCs infected with the different strains of* T. cruzi* presented great similarity. When compared with the other groups, the LPS group also did not show differences in DC apoptosis.

### 3.3. *T. cruzi* Strains Differentially Affect the Production of Pro- and Anti-Inflammatory Cytokines and CCL2

**Figure 3:**
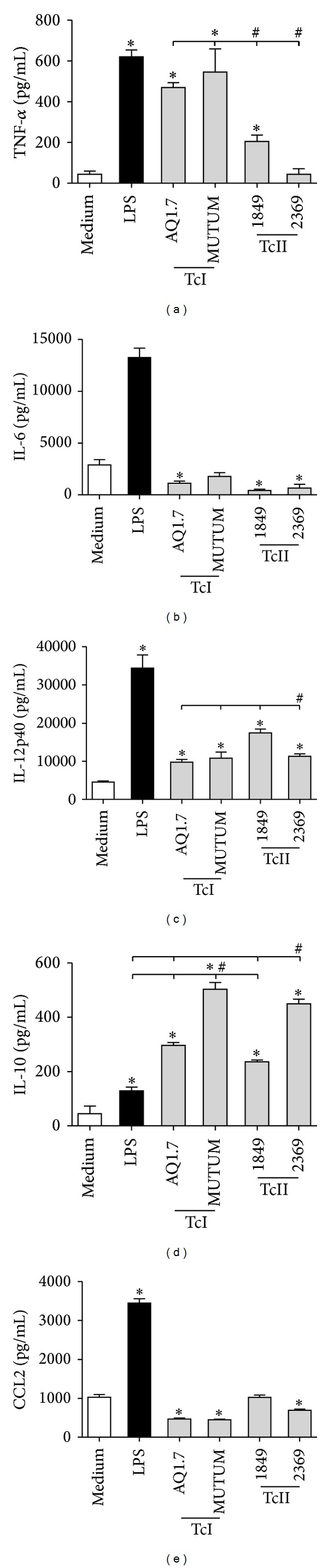
*T. cruzi* strains differentially affect the production of pro- and anti-inflammatory cytokines and CCL2. Murine bone marrow-derived DCs were incubated for 18 h with no stimulation (medium), LPS (100 ng/mL), or different* T. cruzi* strains (MOI 3 : 1), and the production of cytokines and chemokines was evaluated by ELISA. (a) TNF-*α*. (b) IL-6. (c) IL-12p40. (d) IL-10. (e) CCL2. The bars represent the mean, and the vertical lines represent the standard error. *P < 0.05 compared with noninfected DCs (medium). # and lines: *P* < 0.05 comparing the strains; Student's *t*-test or Mann-Whitney test.

**Figure 4:**
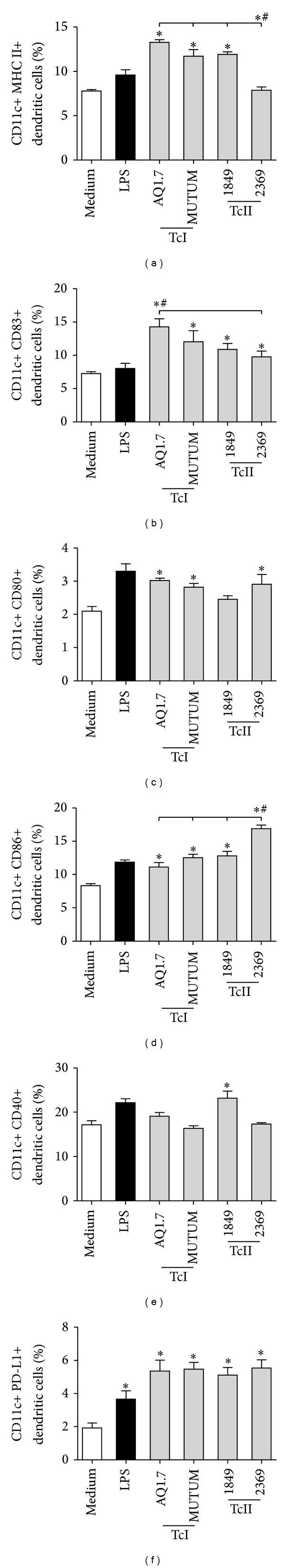
*T. cruzi* strains differentially affect the expression of costimulatory molecules by DCs. Murine bone marrow-derived DCs were incubated for 18 h with no stimulation (medium), LPS (100 ng/mL), or different* T. cruzi* strains (MOI 3 : 1), and costimulatory molecule expression was evaluated by flow cytometry and represented as % of DCs expressing costimulatory molecules. (a) MHC II. (b) CD83. (c) CD80. (d) CD86. (e) CD40. (f) PD-L1. The bars represent the mean, and the vertical lines represent the standard error. *P < 0.05 compared with noninfected DCs (medium). # and lines: *P* < 0.05 comparing the strains; Student's *t*-test or Mann-Whitney test.

DCs were cultured in the presence or absence of the AQ1.7, MUTUM, 1849, and 2369 strains of* T. cruzi*, and their production of IL-6, IL-10, IL-12p40, TNF-*α*, and the chemokine CCL2 was evaluated. In general terms, the results show that DCs infected with different strains of* T. cruzi* were able to produce different patterns of cytokine and chemokine production (Figure 3). LPS was used as a positive control and, as expected, induced high production of the molecules evaluated (Figure 3). When compared with cells that were cultured with medium only, the production of the cytokine TNF-*α* was increased for DCs infected with the strain AQ1.7, MUTUM, or 1849 (Figure 3(a)). The production of TNF-*α* by DCs infected with the 1849 strain was lower than that induced by the AQ1.7 and MUTUM strains. The 2369 strain did not induce significant production of this cytokine (Figure 3(a)). When compared with cells cultured with medium only, the production of IL-6 was reduced in DCs infected with the strain AQ1.7, 1849, or 2369 (Figure 3(b)). The production of IL-12p40 increased in DCs stimulated with any strain, but strain 1849 induced the highest production compared with the other strains (Figure 3(c)). The production of the regulatory cytokine IL-10 by DCs increased when the cells were stimulated with any of the strains studied (Figure 3(d)). However, the MUTUM and 2369 strains induced higher production compared with the AQ1.7 and 1849 strains (Figure 3(d)). Importantly, the production of IL-10 was significantly increased, even when compared with production in cells stimulated with LPS alone (Figure 3(d)). The production of the chemokine CCL2 was reduced in DCs infected with the strain AQ1.7, MUTUM, or 2369 (Figure 4(e)), whereas strain 1849 did not alter this production. It is important to mention that except for TNF-*α*, when DCs were stimulated with different strains of* T. cruzi*, the production of proinflammatory cytokines and CCL2 was much lower compared with production in cells cultured with only LPS or even medium, such as in the cases of IL-6 and CCL2.

### 3.4. *T. cruzi* Strains Differentially Affect the Expression of Costimulatory and Coinhibitory Markers on DCs

An increase in the expression of surface molecules is also observed in DCs that undergo complete maturation and antigen presentation. Knowing that, we evaluated whether DCs infected with different* T. cruzi* strains also have different profiles of expression of the costimulatory and stimulatory markers MHC II, CD83, CD80, CD86, and CD40. Additionally, we evaluated whether the coinhibitory molecule PD-L1 was also modulated by these different strains. In general, the flow cytometric assays demonstrated that DCs infected with the AQ1.7, MUTUM, 1849, or 2369 strain showed different patterns of expression of stimulatory and costimulatory markers on their surface (Figure 4). After 18 h of infection, DCs infected with the AQ1.7, MUTUM, or 1849 strain presented an increased percentage of MHC II expression on their surface, but the AQ1.7 strain induced higher expression compared with the 2369 and 1849 strains (Figure 4(a)). The percentage of expression of CD83 was increased in DCs infected with any strain, and the AQ1.7 strain induced the highest expression compared with the 2369 strain (Figure 4(b)). An increase in the percentage of CD80 was not observed only in DCs that were infected with the strain1839 (Figure 4(c)). The percentage of CD86 was increased in DCs infected with any of the evaluated strains, although the cells infected with the 2369 strain exhibited a more pronounced increase (Figure 4(d)). Only the 1849 strain was able to increase the percentage of expression of CD40 (Figure 4(e)). The results also showed that all strains were able to induce the expression of the PD-L1 coinhibitory molecule after 18 h of DC infection (Figure 4(f)).

### 3.5. *T. cruzi* Strains Differentially Affect the Expression of CCR5 and CCR7 on DCs

**Figure 5:**
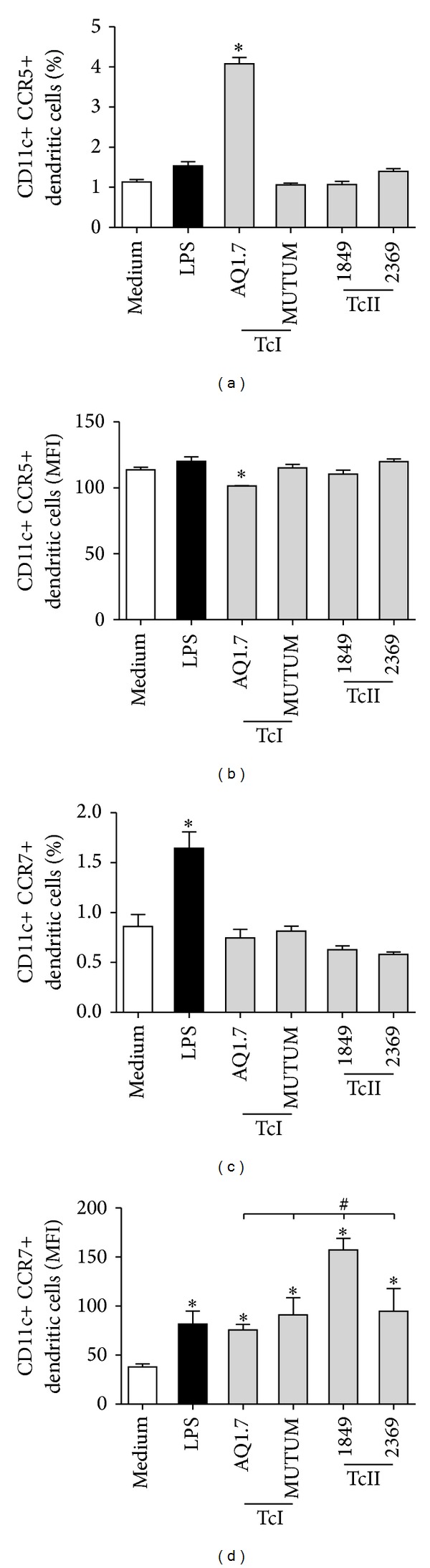
*T. cruzi* strains differentially affect the expression of the chemokine receptors CCR5 and CCR7 in DCs. Murine bone marrow-derived DCs were incubated for 18 h with no stimulation (medium), LPS (100 ng/mL), or different* T. cruzi* strains (MOI 3 : 1), and CCR7 or CCR5 expression was evaluated by flow cytometry. ((a) and (c)) Percentage of DCs expressing CCR5 or CCR7. ((b) and (d)) Intensity of CCR5 or CCR7 expression in DCs based on the MFI. The bars represent the mean, and the vertical lines represent the standard error. *P < 0.05 compared with noninfected DCs (medium). # and lines: *P* < 0.05 comparing the strains; Student's *t*-test or Mann-Whitney test.

To assess whether* T. cruzi* strains modulate these receptors, DCs were incubated with the AQ1.7, MUTUM, 1849, or 2369 strain, and the percentages of expression and the MFIs of these molecules were measured. The results of this experiment demonstrated that DCs infected with the AQ1.7 strain had increased expression of CCR5 (Figure 5(a)). The MFI of CCR5 was practically unchanged in both groups but significantly decreased following infection with the AQ1.7 strain (Figure 5(b)). Regarding CCR7, none of the studied strains modulated the percentage of expression of this receptor (Figure 5(c)). However, the MFI increased for all strains studied, and this effect was more pronounced for the 1849 strain (Figure 5(d)).

### 3.6. *T. cruzi* Strains Differentially Affect the Expression of TLR2 and TLR4 on DCs

**Figure 6:**
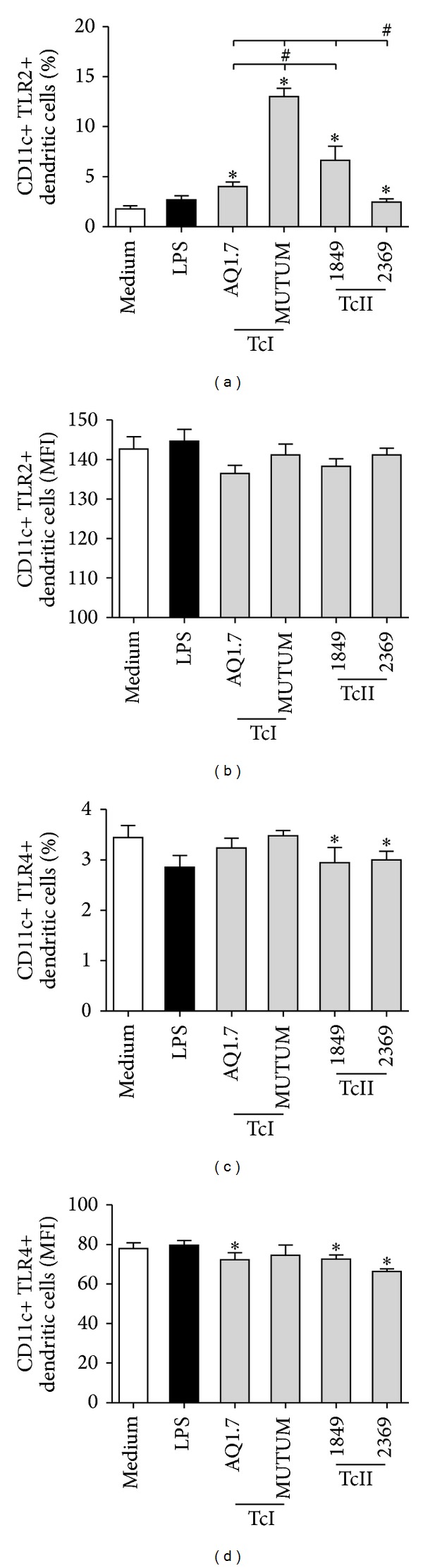
*T. cruzi* strains differentially affect the expression of TLR2 and TLR4 in DCs. Murine bone marrow-derived DCs were incubated for 18 h with no stimulation (medium), LPS (100 ng/mL), or different* T. cruzi* strains (MOI 3 : 1), and TLR2 or TLR4 expression was evaluated by flow cytometry. ((a) and (c)) Percentage of DCs expressing TLR2 or TLR4. ((b) and (d)) Intensity of TLR2 or TLR4 expression in DCs based on the MFI. The bars represent the mean, and the vertical lines represent the standard error. *P < 0.05 compared with noninfected DCs (medium). # and lines: *P* < 0.05 comparing the strains; Mann-Whitney test.

The modulation of the expression and/or activation of innate immune receptors such as TLRs by endo- and ectoparasites have been suggested as a mechanism of immune evasion [24–28]. As the receptors TLR2 and TLR4 have been implicated in the recognition of* T. cruzi* antigens [28, 29], we evaluated whether the different strains of* T. cruzi* are capable of modulating the expression of these receptors. Our results demonstrated that DCs infected with different strains of* T. cruzi* had an increased percentage of expression of TLR2 (Figure 6(a)) and that this increase was most prominent in cells infected with the MUTUM strain. The MFI of TLR2 was not altered by any of the strains tested (Figure 6(b)). DCs infected with strain 1849 or 2369 showed a decrease in the expression of TLR4 compared with noninfected cells (Figure 6(c)). Moreover, the AQ1.7, 1849, and 2369 strains were able to reduce the expression of this receptor (Figure 6(d)).

## 4. Discussion

In this report, it is revealed that four distinct TcI (AQ1.7 and MUTUM) and TcII (1849 and 2369) strains exhibit different rates of infectivity in DCs and that the rate for each strain is weakly related to DC biological and immune parameters. We demonstrate that the TcI strains presented the lowest rates of DC invasion, whereas the TcII strains presented the highest rates. We also show that both the TcI and the TcII strains did not induce significant DC death; however, the production of cytokines, the expression of stimulatory and costimulatory molecules, and the expression of TLRs and chemokine receptors in the DCs varied more between strains than between DTUs themselves. It is truly remarkable that the 2369 strain, from the TcII group, yielded the most interesting results; this strain was the only one that did not induce production of the proinflammatory cytokine TNF-*α*. Additionally, this strain induced high production of the anti-inflammatory cytokine IL-10, among the DC surface molecules, did not induce expression of MHC II and CD40, induced low expression of CD83, and, similar to the other strains, also induced high expression of the coinhibitory molecule PD-L1.

As demonstrated in Figure 1, we show that DC infection rates depend on the infective* T. cruzi* strain because both the percentage and the media intensity of fluorescence of each DC-infecting parasite significantly differed between strains. We believe that this difference is related to the diversity of molecules involved in the entry of* T. cruzi* into the host cells because these molecules vary between* T. cruzi* strains [30–32]. Importantly, none of the strains induced apoptosis or necrosis in the host cells. It is widely known that* T. cruzi* infects all nucleated cells in both experimental animals and humans, although the degree of apoptosis induction in certain host cells requires more detailed studies. Regarding the effect of* T. cruzi* on lymphocyte apoptosis, it is already well known that murine splenic CD4+ and CD8+ T lymphocytes increase the expression of CD95 after infection in mice; this effect is correlated with activation-induced cell death [33, 34]. The treatment of these mice with anti-FasL antibodies protects these mice from death due to* T. cruzi* infection [33, 34]. Furthermore, previous reports have demonstrated that Chagas cardiomyopathy is related to increased apoptosis of cardiomyocytes and augmented Fas-Fas-L expression in situ [35, 36]. Additionally, as the strains studied here induced an increase in PD-L1 expression on DCs, this phenomenon could be a relevant pathway involved in T cell apoptosis [37]. However, concerning other host cells, a recent study showed that* T. cruzi* can also infect and induce apoptosis in macrophages and cardiomyocytes, but this effect is not present in fibroblasts. This ability to infect and induce apoptosis in macrophages and cardiomyocytes is greater in the strains belonging to TcI compared with the TcII strains [38]. Thus, our findings partially differ from this published work because although each strain infects DCs differently, this difference is not related to DC apoptosis. Our results indicate that* T. cruzi* does not induce cell death in DCs. But we believe that the parasite, instead of inducing apoptosis, controls the immune response by manipulating these cells, since the cells remained viable and the pattern of cytokine production and the expression of co- and stimulatory surface molecules were altered. Cells with such characteristics could either induce apoptosis or alter the activation of effector cells.

Knowing that strains of* T. cruzi* invade DCs to different extents, we investigated whether these strains also stimulate the production of cytokines and chemokines and the expression of inhibitory or stimulatory molecules in such cells. Regarding the modulation of the immune response by strains from the same DTU, few results have been published. What we know so far is that, regardless of the strain used, infected patients have an immune response with a proinflammatory profile and that TcI and TcII strains can produce specifically higher levels of certain cytokines compared with other strains [39]. In this sense, it was found that humans infected with TcI strains produce higher levels of the cytokine IL-6, whereas infection with TcII strains induces more IL-1 and IL-17 production. Mixed TcI/TcII infection in patients produces more IL-22 compared with infection with only one strain [39]. In our study, DC chemokine and cytokine production was not DTU dependent because most of the results were similar and, in certain analyses, there were significant differences only between strains from the same DTU. In particular, the production of IL-12 was induced by all strains, and TNF-*α* production was induced by all strains, except for strain 2369, belonging to TcII. The production of IL-6 and CCL2 was similar to or less than production in the cells stimulated with medium only, and the production of IL-10 was increased by all of the strains. However, one of the strains in both the TcI and the TcII groups induced even higher concentrations compared with the other strain in the same DTU. In summary, we suggest that the modulation of cytokine production by these strains is not DTU dependent. In the case of inhibitory and stimulatory molecules, our results showed that DC expression of these molecules was not polarized by different DTUs. The percentages of expression of CD83, CD86, and PD-L1 were increased by all strains. Additionally, the percentages of expression of MHC II and CD80 were not induced by one TcII strain, and the expression of CD40 was enhanced by only one of the TcII strains. Therefore, these in vitro findings show that* T. cruzi* modulates both the production of cytokines and the expression of surface molecules in DCs, but this effect is not DTU dependent but strain dependent. Thus, we believe that the increases in IL-10 and PD-L1 levels, together with the low IL-12 levels and the absence of IL-6 and CCL2 production, may compromise the antigen-presenting and immunostimulatory functions of DCs. These processes may induce immune tolerance, facilitating* T. cruzi* escape from both innate and adaptive immunity.

Prior to activation, DCs present augmented expression of CCR5, but when activated, these cells downregulate CCR5 and overexpress CCR7. This differential expression of these chemokine receptors explains the migration of DCs to peripheral sites or toward inflamed tissues or secondary lymphoid organs, such as the lymph nodes and spleen [40, 41]. Our results showed that only the AQ1.7 strain from TcI was able to increase the percentage of cells expressing CCR5, although these cells present a reduction in expression of this receptor (MFI). In contrast, all of the strains induced the expression of CCR7, and this effect was more pronounced for the 1849 strain from the TcII group. These findings suggest that during* T. cruzi* infection, the traffic of DCs to peripheral tissues is undisturbed or even augmented, such as in the case of the AQ1.7 strain. In the case of CCR7, we presume that the host is able to mature its infected DCs, which migrate to inflamed tissues or the lymph nodes or spleen to present antigens to* T. cruzi*-specific T cells. In this case, our results corroborate the findings that have already been published because it has been demonstrated that chronically* T. cruzi*-infected patients have increased expression of many chemokines and chemokine receptors (including CCR5 and CCR7) in the myocardium and that this phenomenon explains the inflammatory process in the patients after the infection [42]. It is worth mentioning here that Chagas cardiomyopathy manifestations in humans are more correlated with TcI [43]. As CCR5 is a proinflammatory chemokine receptor and the AQ1.7 strain induces more CCR5+ DCs, this receptor could contribute to the inflammatory processes of the patients infected with this strain.

TLRs are involved in both protection against and the pathology of Chagas disease, and among these receptors, it is estimated that TLR2, TLR4, and TLR9 are the most important [26, 44]. Here, we demonstrate that different strains are capable of enhancing the expression of TLR2, and this effect was more pronounced for the MUTUM strain, which belongs to the TcI group. Decreases in the percentage and the mean intensity of TLR4+ DCs were more evident in the strains belonging to TcII. We speculate that the results for TLR2 expression could contribute, in a second moment, to an increased production of IL-10 by infected DCs since* T. cruzi* has molecules that bind to TLR-2 and this and other receptors such as mannose receptors and dectin-1 activate signaling that leads to the production of anti-inflammatory cytokines such as IL-10. Indeed, it is already known that ectoparasites and several species of microorganisms evade the host immune system by inducing the production of IL-10 in a TLR2-dependent manner [25, 26, 45–49]. This phenomenon seemed to have occurred in our study because this increase was very significant. It is known that TLR4 induces large amounts of proinflammatory cytokines, such as IL-12 and TNF-*α*. As the TcII strains induced the lowest production of TNF-*α*, this observation could be explained, even in part, by lower expression of the TLR4 receptor. In fact, it has already been demonstrated in vivo that TLR4 signaling is required for optimal production of IFN-*γ*, TNF-*α*, and nitric oxide (NO) in the spleen of* T. cruzi*-infected animals [26]. Moreover, deglycoinositol phospholipids containing ceramide molecules are recognized by TLR4 and trigger the production of IL-12 and TNF-*α* in macrophages [24, 50]. Thus, decreased expression of this receptor or different amounts of these molecules on the* T. cruzi* surface could explain the lower binding of* T. cruzi* molecules and their consequently lower production.

In summary, we demonstrate, for the first time in protozoa organism, that depending on the strain the parasite may modulate DC biology with different intensities. This observation may have great implications. DCs are essential cells to combat protozoa invaders and trigger antiprotozoa innate and acquired responses so, depending on how they are modulated, the host defense may be completely hampered. Based on our results, it is essential to evaluate the effect of different DTUs of* T. cruzi* in the modulation of the antigen-presenting properties of DCs to T cells so that the patterns of mice immune response are completely changed. These results will corroborate the findings presented here and explain why strains belonging to each population lead to many pathological outcomes associated with acute and chronic Chagas disease. Thus, future studies must be done to test this possibility.

## 5. Conclusions

Taken together, our results demonstrate that TcI and TcII strains of* T. cruzi* may modulate DC biology to different extents. In general terms, whereas strains belonging to both DTUs induce the production and expression of anti-inflammatory molecules, such as IL-10 production and PD-L1 and TLR2 expression, proinflammatory parameters are variably modulated, depending on the strain. These observations suggest that each strain of* T. cruzi* has possibly evolved specific evasion strategies that subvert DCs and consequently the host proinflammatory/immune responses.
